# Gene Dosage of *F5* c.3481C>T Stop-Codon (p.R1161Ter) Switches the Clinical Phenotype from Severe Thrombosis to Recurrent Haemorrhage: Novel Hypotheses for Readthrough Strategy

**DOI:** 10.3390/genes15040432

**Published:** 2024-03-29

**Authors:** Donato Gemmati, Elisabetta D’Aversa, Bianca Antonica, Miriana Grisafi, Francesca Salvatori, Stefano Pizzicotti, Patrizia Pellegatti, Maria Ciccone, Stefano Moratelli, Maria Luisa Serino, Veronica Tisato

**Affiliations:** 1 Department of Translational Medicine, University of Ferrara, 44121 Ferrara, Italy; 2 University Strategic Centre for Studies on Gender Medicine, University of Ferrara, 44121 Ferrara, Italy; 3 Centre Haemostasis & Thrombosis, University of Ferrara, 44121 Ferrara, Italy; 4 Hospital-University of Ferrara, 44121 Ferrara, Italy; 5 Haematology Unit, Hospital-University of Ferrara, 44121 Ferrara, Italy; 6 Laboratory of Technology for Advanced Therapies (LTTA) Centre, University of Ferrara, 44121 Ferrara, Italy

**Keywords:** premature stop-codon (PTC), FV Leiden, cis-segregation, inherited thrombophilia, blood coagulation, readthrough

## Abstract

Inherited defects in the genes of blood coagulation essentially express the severity of the clinical phenotype that is directly correlated to the number of mutated alleles of the candidate leader gene (e.g., heterozygote vs. homozygote) and of possible additional coinherited traits. The *F5* gene, which codes for coagulation factor V (FV), plays a two-faced role in the coagulation cascade, exhibiting both procoagulant and anticoagulant functions. Thus, defects in this gene can be predisposed to either bleeding or thrombosis. A Sanger sequence analysis detected a premature stop-codon in exon 13 of the *F5* gene (c.3481C>T; p.R1161Ter) in several members of a family characterised by low circulating FV levels and contrasting clinical phenotypes. The propositus, a 29 y.o. male affected by recurrent haemorrhages, was homozygous for the *F5* stop-codon and for the *F5* c.1691G>A (p.R506Q; FV-Leiden) inherited from the heterozygous parents, which is suggestive of combined cis-segregation. The homozygous condition of the stop-codon completely abolished the *F5* gene expression in the propositus (FV:Ag < 1%; FV:C < 1%; assessed by ELISA and PT-based one-stage clotting assay respectively), removing, in turn, any chance for FV-Leiden to act as a prothrombotic molecule. His father (57 y.o.), characterised by severe recurrent venous thromboses, underwent a complete molecular thrombophilic screening, revealing a heterozygous *F2* G20210A defect, while his mother (56 y.o.), who was negative for further common coagulation defects, reported fully asymptomatic anamnesis. To dissect these conflicting phenotypes, we performed the ProC^®^Global (Siemens Helthineers) coagulation test aimed at assessing the global pro- and anticoagulant balance of each family member, investigating the responses to the activated protein C (APC) by means of an APC-sensitivity ratio (APC-sr). The propositus had an unexpectedly poor response to APC (APC-sr: 1.09; n.v. > 2.25), and his father and mother had an APC-sr of 1.5 and 2.0, respectively. Although ProC^®^Global prevalently detects the anticoagulant side of FV, the exceptionally low APC-sr of the propositus and his discordant severe–moderate haemorrhagic phenotype could suggest a residual expression of mutated FV p.506QQ through a natural readthrough or possible alternative splicing mechanisms. The coagulation pathway may be physiologically rebalanced through natural and induced strategies, and the described insights might be able to track the design of novel treatment approaches and rebalancing molecules.

## 1. Introduction

Genes expressing blood coagulation factors are coded in proteins belonging to the procoagulant or anticoagulant pathways responsible for the fine-tuning and regulation of those processes that favour haemostasis and contrast thrombosis. Several factors and cofactors possess definite and specialised roles in one or another pathway. Among these, coagulation factor V (FV), encoded by the *F5* gene (1q24.2), is a key component in the rapid activation of prothrombin (FII) to thrombin (FIIa) catalysed by the prothrombinase complex (FXa/FVa) in the procoagulant pathway. Low-circulating FV levels may influence both the laboratory tests and the clinical phenotypes, reserving to the lowest levels, the severest bleeding [1]. At the same time, FV also supports the anticoagulant pathway [2] through the down-regulation of the activated factor VIII (FVIIIa), another key procoagulant cofactor belonging to the tenase complex (FIXa/FVIIIa). In detail, the FV anticoagulant property depends on the enzymatic cleavage of the intact FV by the activated protein C (APC) at a specific FV arginine residue (R506), transforming the procoagulant FV into an anticoagulant protein (FVc > FVac), completely losing any procoagulant asset and favouring the inactivation steps of FVIIIa by the APC, protein S, and the FVac complex (APC/PS/FVac). Apart from the thrombin-mediated cleavage at specific arginine residues (R709, R1018, and R1545) required for FV activation (i.e., FV > FVa) [3,4], other crucial arginine residues (R306, R506, and R679) are necessary for FVa inactivation (i.e., FVa > FVai) through the APC/PS complex [5]. This fine-tuning principally depends on the local concentration of procoagulant and anticoagulant enzymes that specifically decide which side of FV prevails, and the coexistence of additional inherited predispositions in the genome of the patient may contribute to the establishment of the final clinical phenotype [1,2].

*F5* gene mutations, which are responsible for FV deficiencies, cause a rare haemorrhagic coagulation disorder with an incidence of about 1:1,000,000. They are inherited as autosomal recessive traits, and the clinical manifestations range from asymptomatic to severe bleeding, in which homozygosity or compound heterozygosity assigns the severest phenotypes [6,7,8]. Several mutations in the *F5* gene responsible for FV deficiencies have been reported [9,10,11,12,13,14,15] in a wide database that also reports functional variants [16]. A few of them represent recurrent mutations, such as p.Y1702C in the Italian population [13,17] and p.R1161Ter, a premature termination codon (PTC) with no geographical clustering [18,19,20,21,22,23]. In heterozygosis they cause the classical type I deficiency characterized by mild phenotypes and 50% of procoagulant activity, whilst the homozygosis causes severe to moderate bleeding. Instead, the majority of *F5* mutations have been found in unique families and considered as private mutations [22].

*F5* gene mutations, playing a major role in thrombosis, ascribe the greatest role to Factor V Leiden (c.1691G>A). The mutation results in an arginine-to-glutamine substitution in one of the most functional APC cleavage sites in FVa (p.R506Q), causing the well-known inherited APC-resistant phenotype [24,25]. Heterozygotes have a five- to seven-folds increased risk of venous thromboembolism, and the risk is exponentially higher in homozygous carriers (about 80-folds), with it being the most frequent genetic risk factor for venous thrombosis in Caucasians [24,25]. Additional common and less common *F5* gene mutations or polymorphisms may coexist in cis or trans with a leader mutation, exacerbating the clinical phenotype as well as the circulating FV levels (e.g., *F5* p.Y1702C and *F5* p.M2148T) or the anticoagulant activity via an APC-resistant phenotype (e.g., FV Leiden, FV HR2, FV Cambridge, and FV Hong-Kong) [1,2,26,27,28,29,30,31]. Therefore, according to the role of the leader mutation in the coagulation balance, the coexistence of additional variants may have synergic or antagonistic effects, resulting in different clinical phenotypes.

FV Leiden and PTCs can be inherited on the same allele (cis) or on different alleles (trans), leading to a pseudo-normal or a pseudo-homozygous phenotype, respectively [13,32,33]. Accordingly, the probability of carrying one or both defects drastically changes in the two models. Additionally, cis inheritance wipes out any chance for the FV allele to be expressed, changing, in turn, the clinical phenotype of the carrier patients. The research hypothesis stems from the observation that the same leader mutation can cause contrasting clinical phenotypes by exacerbating or smoothing the phenotypes of possible additional coinherited defects in the same individual. Moreover, the dualistic role of FV assigns to this molecule the possibility to change the phenotype in both directions, acting in synergy or in antagonism with the ancillary defects.

Interestingly, in a previous study performed by our group, we concluded that extended investigations on additional coagulation gene defects selected by GWAS [34] could benefit large families with unexplained thrombotic members, while in the presence of well-characterised causative mutations, this approach scarcely improved the final diagnosis.

Therefore, in the present investigation, we discuss and report a family in which the same leader mutation (i.e., *F5*, p.R1161Ter), in combination with different individual genetic backgrounds of the members, drastically drives the clinical phenotype of the carriers from completely asymptomatic to severe thromboembolism until recurrent haemorrhage. The genotype–phenotype comparison of the data that arose from this study confirms the polygenic and multifactorial regulation of blood coagulation, paving the way for novel alternative therapeutic strategies.

## 2. Materials and Methods

### 2.1. Blood Sample Collection and DNA Extraction

The family investigated in this report belongs to a local project entitled “Multilocus Genetic Scores predictive for Venous Thromboembolism Risk (MaGiSTER): real life evaluation and validation in a cohort of VTE patients”, which is aimed at scoring VTE risk modifications within thrombophilic families characterised by well-established gene defects [34]. The MaGiSTER study was approved by the local IRB (code number 242/2020) according to the Helsinki Declaration, and all the members of the investigated family signed informed consent at the time their blood was drawn.

Whole blood samples were collected in sodium citrate anticoagulant and centrifuged at 2000× *g* for 15 min, and plasma was immediately stored at −25 °C. Genomic DNA was isolated from the frozen blood using an automated DNA extraction and purification robot, the BioRobot EZ1 system (Qiagen, Hilden, Germany). The DNA was quantified using a spectrophotometer (Agilent Technologies, Santa Clara, CA, USA).

### 2.2. PCR Amplification and Sanger Sequencing

PCR amplification of the coding regions of the *F5* gene was performed on Agilent SureCycler 8800 (Agilent Technologies, Santa Clara, CA, USA) in a 50 μL reaction containing 300 ng of template DNA, 2.5 U of AllTaq DNA polymerase (AllTaq PCR Core and Master Mix Kits, Qiagen) and 0.25 μM of forward and reverse primers for each specific exon, as previously described [35,36]. In detail, primers matching exons 10 and 13 have been modified, as reported in Table 1.

Amplicons were purified by a PureLink PCR Purification kit (Invitrogen, Thermo Fisher Scientific, Waltham, MA, USA) and sequenced using a ProDye Terminator Sequencing System kit (Promega Corporation, Madison, WI, USA). The reaction mixture (20 µL) contained 50 ng of purified amplicons, a Master Mix and Sequencing Buffer, and 5 pmol of forward or reverse primer. The sequencing products were precipitated in ethanol-EDTA, and resuspended in Hi-Di Formamide, capillary electrophoresed on a Spectrum Compact CE System (Promega Corporation, Madison, WI, USA), and finally analysed by BioEdit analysis software (Informer Technologies, Inc., v. 7.7.1, Garden Grove, CA, USA).

### 2.3. Global and Specific Anticoagulant Response to APC

The in vitro assay was the APTT-based method (ProC^®^ Global, Siemens Helthineers, Forchheim, Germany), which was modified, as previously described [37]. The assay measures the elongation time (s) of the basal APTT obtained in the presence of the PC activator (Protac^®^, Siemens Helthineers, Forchheim, Germany). In detail, it was performed in the presence or absence of the PC activator (Protac^®^, Siemens Helthineers, Forchheim, Germany) by adding one volume for each of the following reagents: undiluted plasma sample; plus activator (APTT + Protac^®^) or buffer (APTT—Protac^®^); APTT reagent (Pathromtin^®^SL, Siemens Helthineers, Forchheim, Germany); activation time (180 s at 37 °C); CaCl_2_ (25 mM). The results were expressed as an APC sensitivity ratio (APC-sr) = [(APTT_sec_ + Protac^®^): (APTT_sec_—Protac^®^)] of the test plasma, representing the folds of the elongation time of the basal APTT (s) obtained in the presence/absence of the PC activator. An elongation time value of APC-sr > 2.25 folds was considered normal (the lowest limit defined as the 10th percentile of 110 normal subjects). To improve sensitivity and have more specific assessments of the anticoagulant power of FV compared to the classical anticoagulant molecules (PC and PS) or a classical procoagulant molecule (FVIII), scalar dilutions (range 0–100%) of normal pool plasma in different PC-, PS-, FV-, or FVIII- immune-depleted plasmas (Siemens-Healthineers, Forchheim, Germany) were tested as described above and as previously reported by our group [37,38,39]. In addition, FV-Leiden scalar dilutions (0–100%) were obtained by mixing normal pool plasma or FV-immune-depleted plasma with a pool of 10 different subjects homozygous for the FV p.R506Q mutation, which was also assessed for having normal levels of FV in the ACL 7000 automated coagulometer (Instrumentation Laboratory, Werfen-Italia, Italy). Similarly, the undiluted plasma of the three members of the family have been tested, and the elongation folds in the presence/absence of the PC activator (Protac^®^) are shown as APC-sr [37,40].

## 3. Results

### 3.1. Family Characteristics and Clinical and Laboratory Phenotypic Findings

**Figure 1:**
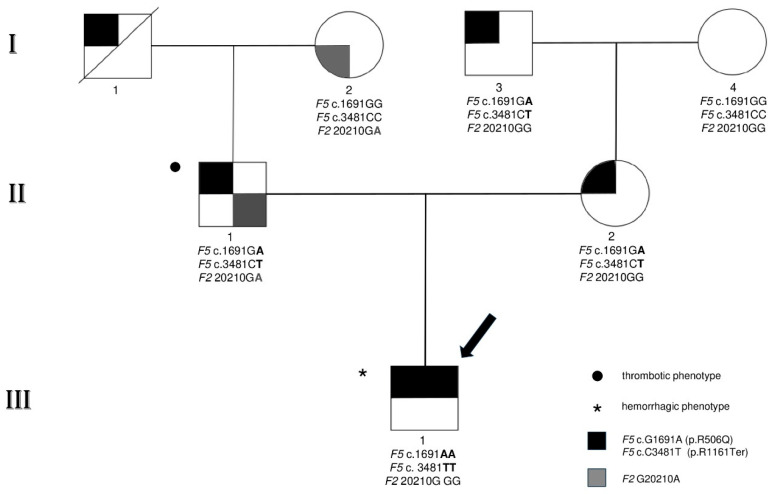
Full pedigree of the original family. Black symbols refer to the combined *F5* cis-defect (i.e., p.R506Q; p.R1161Ter); grey symbols refer to *F2* G20210A substitution. The asterisk indicates the haemorrhagic phenotype (**III1**), the black circle the thrombotic phenotype (**II1**). Strike-through symbol indicates a dead individual (**I1**). Laboratory analyses in subject (**I1**) have not been determined due to his death at the time of investigation; nevertheless, the *F5* cis-heterozygosity (i.e., p.R506Q; p.R1161Ter) has been shown after excluding this haplotype in his wife (**I2**). The arrow indicates the index patient (i.e., propositus). (**I3**), (**I4**) and (**II2**) did not report clinical manifestations ascribable to a positive history of coagulopathy, nevertheless subjects I3 and I2 carry the *F5* cis-defect (i.e., p.R506Q; p.R1161Ter) and the *F2* G20210A respectively.

We report a family carrying a stop-codon in the *F5* gene (i.e., p.R1161Ter), characterised by extreme opposite clinical manifestations affecting the pathophysiology of blood coagulation on different sides (Figure 1).

The clinical phenotypes in the family accounted for relapsing severe thromboembolic events in the father (II1), a complete asymptomatic anamnesis in the mother (II2), and recurrent haemorrhage episodes in their son (III1). The remaining individuals, shown in Figure 1, did not report clinical manifestations ascribable to a positive history of coagulopathy. It is worth noting that *F5* p.R1161Ter has been recently investigated in the propositus through molecular approaches to test if the stop-codon could be responsive to in vitro and ex vivo rescues by readthrough strategies [23].

In detail, the propositus (III1), a 29 y.o. male with a previous medical history of recurrent bleeding, was diagnosed at the age of three after a traumatic haemorrhage with a severe FV deficiency with undetectable circulating factor levels (FV:C < 1%, FV:Ag < 1%). His parents reported that he had been suffering from easy bruising since early infancy, with a bleeding history including recurrent knee haemarthroses from the age of nine, an episode of rectal bleeding at the age of 20, and one episode of haematoma in the right thigh muscle at the age of 21. Given that FV concentrate was not commercially available and that it was undergoing preliminary testing, the patient had been treated on demand with fresh-frozen plasma and human-recombinant-activated FVIIa (NovoSeven RT, Novo Nordisk, Malmo, Sweden). The main molecular mechanism responsible for the above-described clinical phenotype is an ineffective activation of prothrombin (FII) to thrombin (FIIa), catalysed by the prothrombinase complex in the procoagulant pathway. The lack of FVa cofactor activity (i.e., homozygous *F5* p.R1161Ter) dramatically affects thrombin generation, leading to unsuccessful haemostatic power and poor fibrin plug formation with consequent recurrent haemorrhages in the propositus.

The father (II1) experienced their first episode of deep-vein thrombosis and a massive pulmonary embolism at the age of 32 years after a long-distance car journey, followed by successive recurrent episodes of superficial thrombophlebitis despite prompt oral anticoagulant therapy (i.e., vitamin K-antagonist; Coumadin). At present, he is on life-long treatment with direct anticoagulants (DOACs; Apixaban, 2.5 mg twice a day). The laboratory thrombophilia screening revealed a reduced response to APC, which was confirmed by low APC-sr values.

A more complex molecular mechanism affects the anticoagulant side in the father. In this case, low anticoagulant activity of FVac, which is mainly due to low levels of FV caused by the combined heterozygosis, *F5* p.R1161Ter and *F5* p.M2148T on different alleles, does not properly counteract the prothrombotic action of the gain of function *F2* G20210A. Unrestrained prothrombin activity, exacerbated by high-circulating levels (i.e., FII:C, 130%), can lead to recurrent thrombotic episodes in the father.

Conversely, the mother (II2) did not experience episodes of thrombosis or haemorrhage during her life despite reduced FV activity. In the absence of additional unbalancing defects, this confirms an efficient haemostatic competence of FV near 50%.

Both parents had FV levels matching a type I deficiency (father II1, FV:C, 38%, and mother II2, FV:C, 50%), compatible with the presence of one copy of a null-functional *F5* allele in their genotype, which was then coinherited in a double copy by their son, accounting for his undetectable FV levels (Table 2).

### 3.2. Genetic Investigations

**Figure 2:**
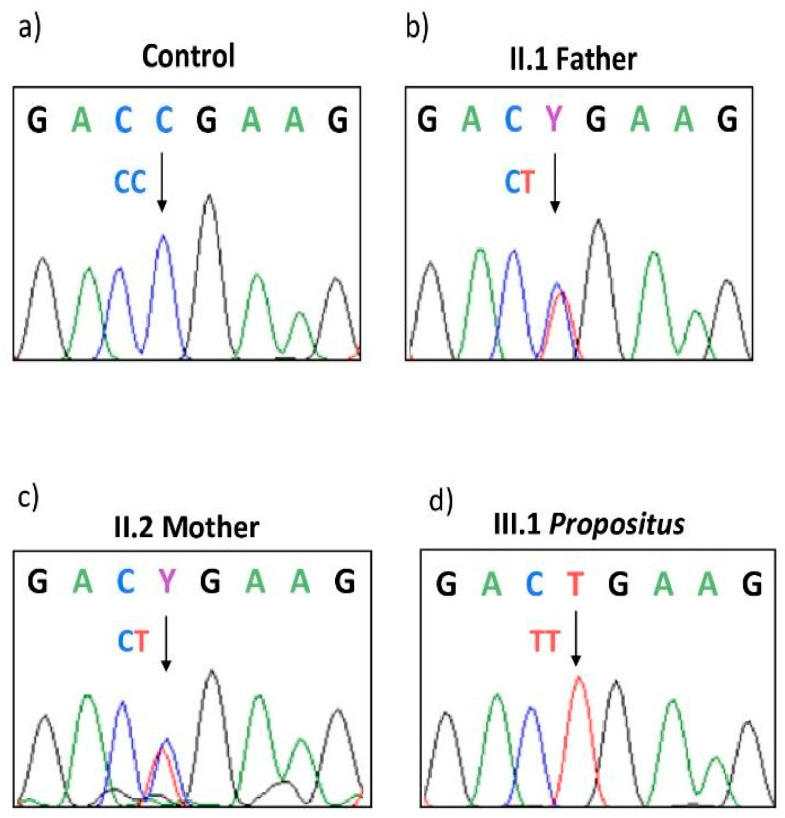
Sanger sequencing in *F5* exon 13 showing the c.3481C>T stop-codon. (**a**–**d**) normal control, heterozygous pattern in the father (**II1**) and in the mother (**II2**), and homozygous status in the propositus (**III1**) respectively. In the nucleotide sequence, Y indicates that both C and T have been detected.

Sanger sequence analysis in the members of the family detected a c.3481C>T mutation (CGA>TGA) in exon 13 of the *F5* gene (Figure 2), causing a non-functional allele due to a PTC at arginine 1161 (p.R1161Ter), and a c.1691G>A transition (rs6025) in exon 10, causing an arginine-to-glutamine change in codon 506 of the FV molecule (p.R506Q; FV-Leiden). The two defects, segregated in cis, and were inherited by the homozygous son (III1) from both the heterozygous parents, causing a double copy of a strong pro-haemorrhagic phenotype due to the lack of circulating procoagulant FV. On the other hand, homozygous FV Leiden (p.R506Q) could not express its strong prothrombotic status since it vanished due to the stop-codon cis-segregation. In addition, the father (II1) was also a carrier of c.6443T>C (rs9332701) in the unique functional *F5* allele, causing a methionine-to-threonine change at codon p.M2148T, which has previously been reported as causative of reduced FV levels [41]. This explains his lower FV level than that theoretically predicted by a heterozygous type I gene defect, as was found in the mother (II2). Finally, the father was heterozygous for *F2* G20210A (rs1799963) in the prothrombin gene, seriously unbalancing the anticoagulant pathway in the context of his peculiar coagulative genetic landscape.

### 3.3. In Vitro Assessment of the Anticoagulant Power of FV by APC-sr

To measure precisely and compare the procoagulant and anticoagulant degree of each molecule involved in the APC/PS system and to weigh the effective role of the mutated residual FV molecule in the family members, we assessed the global anticoagulant response to APC using a modified APTT-based ProC^®^ Global test (Siemens-Healthineers, Forchheim, Germany), as described in the Section 2.

**Figure 3:**
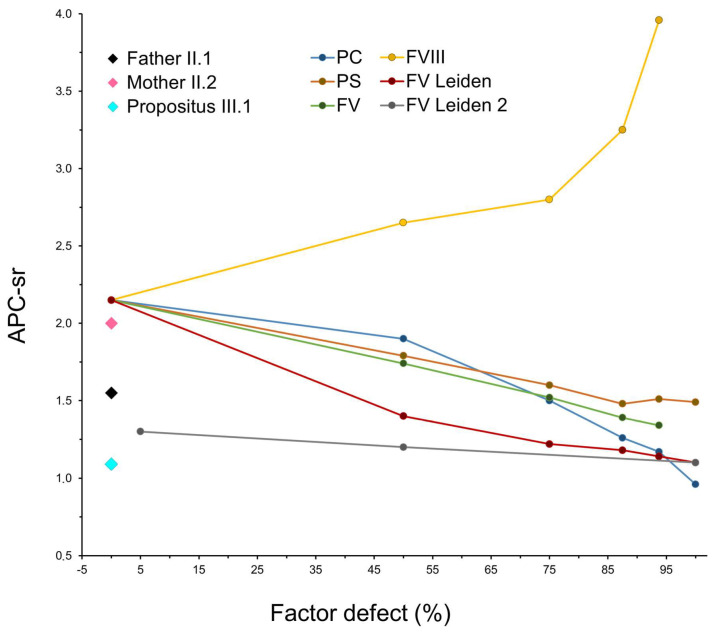
Anticoagulant response of PC, PS and FV assessed as ratio of sensitivity to APC (APC-sr) of different scalar dilutions (0–100%) in comparison to that of FV-Leiden and FVIII. Black, pink and light-blue diamonds (on the left), show the undiluted APC sensitivity ratios of the three family members (II1, II2, and III1, respectively). “FV Leiden” indicates FV 506QQ sample dilution with normal pool plasma, and “FV Leiden 2” indicates FV 506QQ sample dilution in FV-immune-depleted plasma as detailed in the Methods section. Note that, to obtain measurable APTT seconds, the lowest proportion of “FV Leiden 2” in FV-deficient plasma starts at 5% of factor defect (%), similarly the highest dilution of “FV” ends at 95% of factor defect (%), this because both samples cannot yield measurable APTT seconds at FV concentration below 5%. The remaining samples are in the full range (0–100%).

Figure 3 shows the anticoagulant profiles of PC, PS, and FV assessed as the ratio of sensitivity to APC (APC-sr) of the different scalar dilutions and compares them to FV-Leiden (a reference APC-resistant molecule) and FVIII (a classical factor showing only procoagulant ability). In detail, it is strongly evident that, as the percentage of FV-Leiden increased in the system, the APC-sr score decreased, and so they are inversely related (red line in Figure 3). On the contrary, APC-sr and FVIII levels (assessed as a percentage of the defect) are directly related, which means that as the levels of FVIII decreased, the response and sensitivity to APC increased (yellow line in Figure 3). Interestingly, when the FV-Leiden sample was diluted with FV-depleted plasma (grey line in Figure 3), it showed a more resistant phenotype in terms of APC-sr compared to the dilutions performed in the normal pool plasma (red line). This is in accordance with the reported pseudo-homozygous condition of FV-Leiden, which was established when p.R506Q and an FV type I deficiency have trans-coinheritance [32,33].

Interestingly, APC-sr values decreased as the amount of normal FV decreased in the in vitro system (green line in Figure 3), showing that the comprehensive action of the FV molecule is the result of two components, computable as FV-anticoagulant > FV-procoagulant (FVac > FVc), with an overall prevalence on the anticoagulant side. As expected, PC maintained a progressive reduction in APC-sr values at all dilutions, reaching the lowest ratio at 0% of the molecule present in the in vitro system (blue line). Although PS scalar dilutions resemble the trend observed in PC, they reached a plateau at around 10% of residual PS activity (brown line). Finally, Figure 3 also shows the undiluted APC sensitivity ratios of the three members of the family (Table 2), strongly reduced in the propositus (APC-sr: 1.09), reduced in the father (APC-sr: 1.55), and borderline in the mother (APC-sr: 2.0); here, we considered normal APTT elongation values > 2.25 folds.

## 4. Discussion

Inherited haemorrhagic disorders such as haemophilia follow the single-gene defect model and are good candidates for gene therapy, unlike inherited thrombophilia, which, indeed, has a complex polygenic architecture [42,43]. Accordingly, the wide variability in the clinical manifestations and laboratory phenotypes of familial thrombophilia [34,38,39,40,44,45] drastically declines in classical haemophilia, though rare bleeding disorders or combined defects, or the presence of circulating dysfunctional molecules can contribute to a sort of heterogeneity that also affects the bleeding tendency and treatment efficacy [46,47].

Defects in blood coagulation due to disturbances in the same gene pathway could potentially manifest opposite clinical phenotypes when also considering the same gene. This is because gain- or loss-of-function mutations, in affecting the natural anticoagulant or procoagulant factors, may deregulate the global coagulation homeostasis, acting as either protective or risk factors, leading to different imbalances [48]. Considering that the severity of a phenotype or the risk of developing a manifest clinical phenotype increases as a result of the number of mutated alleles in the genotype of carriers (e.g., wild-type, heterozygote, or mutated homozygote), the extent to which a gene dosage may induce a particular phenotype is essentially due to the expression level of the remaining functional allele, the individual genetic/acquired context (i.e., the coexistence of additional defects), and the pattern of inheritance of the leader defect (e.g., dominant or recessive).

In the case of the *F5* gene, it is coded for a product with a dualistic role, the *Janus-faced* protein, as Dahlbäck called it in 2002 [49]. Low levels of FV primarily affect the procoagulant side, causing haemorrhages in the case of severe defects, whilst a mild/moderate deficiency is often asymptomatic, contributing, furthermore, to the weakening of the anticoagulant side, thus favouring thrombosis [1,2,50]. This genetic-based model might be analogous to a complex and challenging complication in blood coagulation called disseminated intravascular coagulation (DIC). The coexistence of thrombosis and haemorrhages due to factor consumption/shortage assigns to DIC peculiar characteristics, revealing the double vulnerability of a coagulation balance [51,52]. Similarly, in our research, these concepts perfectly match and adequately paint the multifaceted architecture of haemostasis and thrombosis.

Here, we described a family in which a PTC caused contrasting clinical phenotypes according to the *F5* gene dosage. The father (II1) experienced severe thromboses caused by a detrimental genetic background characterised by at least one full-blown prothrombotic condition (i.e., *F2* G20210A), which was further compromised by reduced anticoagulant FV-cofactor action (FVac). The low anticoagulant activity of FVac is strictly related to the low-FV-circulating levels (FV:C, 38%) and is exclusively sustained by the only active allele [i.e., *F5* R1161Ter (null allele) and *F5* M2148T (active allele)]. This condition does not properly counteract the prothrombotic action of *F2* G20210A; therefore, an unrestrained prothrombin activity exacerbated by high-circulating levels (i.e., FII:C, 130%; n.v. 70–125%) led to recurrent thrombotic episodes in the father.

The son (III1) experienced spontaneous bleeding and recurrent haemorrhages after trauma caused by a severe deficiency of FV, mainly compromising procoagulant FV-cofactor action in the prothrombinase complex (i.e., homozygous *F5* stop-codon). This caused an ineffective activation of prothrombin to thrombin (FII > FIIa) in the procoagulant pathway, dramatically affecting thrombin generation with the consequence of unsuccessful haemostatic power and a poor fibrin plug formation, with recurrent haemorrhages in the propositus.

The mother (II2) was completely asymptomatic and negative for past and present personal history of any coagulopathy, and this was also the case after surgery, despite a reduced amount of FV (FV:C, 50%). These levels are still enough to guarantee sufficient procoagulant FV cofactor activity in the prothrombinase complex and sufficient anticoagulant FV cofactor activity in the APC/PS complex in the absence of concomitant-inherited defects.

The combination of identified genetic alterations may only partially explain what happens at the molecular level. Therefore, to dissect these opposing genotype–phenotype associations, we assessed the procoagulant and anticoagulant burden of each member of the family and provided a detailed evaluation of the net contribution of the different components of the APC/PS system (i.e., PC, PS, and FV). The in vitro system ascribed to the propositus an anomalous and unexpected low sensitivity to the APC, which is essentially imputable to the lack of FVac ability in the patient’s plasma. Although in contrast with the clinical haemorrhagic phenotype of the propositus, it is in line with the decreased APC-sr obtained as the amount of normal FV decreased in the in vitro system. This allowed us to assign, at least in vitro, a comprehensive function computable as FV-anticoagulant > FV-procoagulant (FVac > FVc) with prevalence on the anticoagulant side. On the other hand, this result is strengthened and validated by the increased APC-sr in the presence of low FVIII levels, which is representative of the high sensitivity and capability of the test to evaluate more permissive APC responses that are contingent on the different molecules considered. It is important to note that FV and FVIII have strong procoagulant activity in vivo and in vitro, and while FVIII has mere procoagulant activity, FV has both, which is the result of the combined functions.

The exceptionally low APC-sr of the propositus and his moderate–severe haemorrhagic phenotype could suggest a residual expression of the mutated FV p.506QQ due to a natural readthrough or possible alternative splicing mechanisms. Afterwards, we could not exclude that in vivo residual FV-mutated mRNA could be expressed as full-length and/or as short-FV molecule(s) due to PTC escape mechanisms, low nonsense-mediated decay pathway (NMD), or alternative splicing events via a natural readthrough or FV-short isoforms, as recently suggested by Castoldi et al. [23,53]. These actions may have significant effects on the global pro- and anticoagulant pathways of carrier patients, considering that in the members of this family, p.R1161Ter was cis-linked with the prothrombotic p.R506Q mutation. This may speculatively be helpful in the bleeding mitigation of the FV severe deficiency via the internalisation and replenishing of FV in platelet α-granule, prolonging, in turn, circulating FV half-life in the plasma [54,55].

On the other hand, we cannot exclude the existence of additional prothrombotic defects in the father (a part *F2* G20210A) might contribute to his severe thrombotic phenotype. In the same way, we cannot ignore possible additional compensatory actions in the coagulation balance that could ameliorate the severe FV deficiency in the propositus. Accordingly, the moderate-bleeding phenotype could be theoretically supported by other low-anticoagulant pathways, such as a TFPI (Tissue Factor Pathway Inhibitor), and further exacerbated in the propositus by the absence of FV, which is the main TFPI protective carrier [56,57].

Through the present methodology, we deepened a specific aspect of the coagulation cascade, even though its complex hierarchical nature deserves a multilayer omics approach [58] involving genomics, transcriptomics, and proteomics. In the last few years, the study of individual molecules has been empowered by “systems biology”. This concept seeks to provide a quantitative framework to understand blood as a reactive biological fluid whose function is dictated by prevailing pathway kinetics, haemodynamics, vessel wall characteristics, platelet metabolism, and small molecules released during coagulation beyond coagulation factors in plasma [59]. The blood coagulation cascade is a perfect paradigm for system biology due to its positive and negative complex connections with other pathways, such as complement innate immunity and inflammation. Advanced technological tools of systems biology, e.g., the Hockin–Mann model [60] and predictive computational simulations [59], can come to our aid for this purpose. Nowadays, they are fundamental to modern clinical research and the subsequent development of targeted drugs. Systems medicine can shed light on multiple research scenarios, ultimately leading to the practical result of uncovering dynamic interaction networks that are critical for influencing the course of medical phenotypes and patient-specific therapeutic measures [61].

## 5. Conclusions

This investigation clearly depicts the intrinsic complex nature of the physiopathology of blood coagulation, its polygenic and multifactorial homeostasis, and its regulation that are mainly determined by the coinheritance of additional defects or traits. Considering that the blood coagulation cascade may be rebalanced or further unbalanced by the inhibition or activation of parallel specific pathways, this could help the design of novel treatment strategies or targeted molecules.

The propositus moderate haemorrhagic phenotype might be related to a residual expression of the mutated FV p.506QQ that is derived from a natural readthrough [23,53]. This event makes the translational machinery recode the PTC, promoting the accommodation of near-cognate aminoacyl-tRNAs in the ribosomal acceptor site due to a fidelity reduction of the contingent protein synthesis [62]. As a result, novel amino acid insertions allow an alternative reading frame to generate minimal percentages of full-length functional protein [63].

A natural readthrough translation can be desirable when a PTC is found in a critical gene [64], although the probability is too low to provide any effective benefit. For this reason, a pharmacologically induced readthrough may be eligible to increase the natural readthrough, and coagulation deficiencies represent an ideal target. In situations where FV levels are severely depleted, the potential rescue of protein expressions by 1–5% may prove to be crucial [23,53] in achieving sufficient haemostasis. In fact, the employment of readthrough-inducing compounds has already been shown to be efficient in coagulation defects. For example, Ataluren (PTC-124) treatment is actually in phase II clinical trials for Haemophilia A and B (NCT00947193).

Growing evidence reveals that the choice of a proper readthrough molecule should take into account the individual molecular architecture, which is the core concept of precision medicine [65]. The genetic context of the stop-codon, the local mRNA topology, and the molecules that interact with the mRNA region downstream to the PTC determine the global readthrough efficiency [65].

The present stepping-stone investigation lays the foundation for strictly personalised medicine. Theoretically, CGA>TGA (p.R1161Ter) is preferentially recoded by spontaneous readthrough compared to other conventional stop-codons (UAG or UAA). In addition, the presence of a purine in the +4NT position immediately following the stop-codon results in a more efficient “leakiness” of PTC compared to pyrimidines [66].

Considering the relevant impact of the individual complex molecular context on readthrough success, the future direction of the present research will be to design a personalised induced-readthrough strategy. This, by selecting low-toxic novel classes of nonsense suppressor drugs (e.g., ELX-02 and PTI-428) and/or molecules as guanidino quinazolines and pyrimidines scaffolds [53,67,68], together with tRNAs and antisense oligonucleotides, provide a selective tropism towards a desired cell or tissue [69,70].

Further successful strategies could be the synergic combination of induced-readthrough agents with suppressors of the NMD pathway [71,72] and the creation of a model reflecting the global patient peculiarities represented by the induced pluripotent stem cells (iPSCs), which are powerful preclinical models to test novel readthrough-selected molecules.

## Figures and Tables

**Table 1:** Primer sequences for PCR-based analyses of *F5* exons 10 and 13.

*F5* Gene	Exon	FV Protein	SNP	Primer Sequence	Size (bp)
c.1691G>A	10	p.R506Q	rs6025	Fw: CATACTACAGTGACGTGGAC	206
				Rv: TGTTCTCTTGAAGGAAATGC	
c.3481C>T	13	p.R1161Ter	rs118203909	Fw: GACACTGGTCAGGCAAGCTG	307
				Rv: TGAGGTCTGGAGAGAGGTTTGT	

**Table 2:** Main molecular and coagulation laboratory findings within the family members.

Family Members	*F5* c.3481 C>T	*F5* c.1691G>A	*F2* G20210A	FV:C (%)	APC-sr
I1	C**T**	G**A**	GG	n.e.	n.e.
I2	CC	GG	G**A**	115	2.9
I3	C**T**	G**A**	GG	**48**	**2.1**
I4	CC	GG	GG	110	3.1
II1	C**T**	G**A**	G**A**	**38**	**1.55**
II2	C**T**	G**A**	GG	**50**	**2.0**
III1	**TT**	**AA**	GG	**<1**	**1.09**

In bold, the polymorphic alleles and the pathologic values; FV:C, n.v.: 70–125%; APC-sr, n.v. > 2.25; n.v. stands for normal values; n.e. stands for not evaluated.

## Data Availability

The original contributions presented in the study are included in the article, further inquiries can be directed to the corresponding author.
